# ^13^C tracer analysis reveals the landscape of metabolic checkpoints in human CD8^+^ T cell differentiation and exhaustion

**DOI:** 10.3389/fimmu.2023.1267816

**Published:** 2023-10-19

**Authors:** Alexander Kirchmair, Niloofar Nemati, Giorgia Lamberti, Marcel Trefny, Anne Krogsdam, Anita Siller, Paul Hörtnagl, Petra Schumacher, Sieghart Sopper, Adolf Sandbichler, Alfred Zippelius, Bart Ghesquière, Zlatko Trajanoski

**Affiliations:** ^1^ Institute of Bioinformatics, Biocenter, Medical University of Innsbruck, Innsbruck, Austria; ^2^ Department of Biomedicine, Cancer Immunology, University and University Hospital of Basel, Basel, Switzerland; ^3^ NGS Core Facility, Biocenter, Medical University of Innsbruck, Innsbruck, Austria; ^4^ Central Institute for Blood Transfusion and Immunology, Tirol Kliniken GmbH, Innsbruck, Austria; ^5^ Core Facility FACS Sorting, University Clinic for Internal Medicine V, Medical University of Innsbruck, Innsbruck, Austria; ^6^ Institute of Zoology, University of Innsbruck, Innsbruck, Austria; ^7^ Laboratory of Applied Mass Spectrometry, Department of Cellular and Molecular Medicine, KU Leuven, Leuven, Belgium; ^8^ Metabolomics Core Facility Leuven, Center for Cancer Biology, VIB, Leuven, Belgium

**Keywords:** 13C tracer analysis, immunometabolism, RNA sequencing, differentiation, stem cell memory cells, exhaustion

## Abstract

**Introduction:**

Naïve T cells remain in an actively maintained state of quiescence until activation by antigenic signals, upon which they start to proliferate and generate effector cells to initiate a functional immune response. Metabolic reprogramming is essential to meet the biosynthetic demands of the differentiation process, and failure to do so can promote the development of hypofunctional exhausted T cells.

**Methods:**

Here we used 13C metabolomics and transcriptomics to study the metabolism of CD8+ T cells in their complete course of differentiation from naïve over stem-like memory to effector cells and in exhaustion-inducing conditions.

**Results:**

The quiescence of naïve T cells was evident in a profound suppression of glucose oxidation and a decreased expression of ENO1, downstream of which no glycolytic flux was detectable. Moreover, TCA cycle activity was low in naïve T cells and associated with a downregulation of SDH subunits. Upon stimulation and exit from quiescence, the initiation of cell growth and proliferation was accompanied by differential expression of metabolic enzymes and metabolic reprogramming towards aerobic glycolysis with high rates of nutrient uptake, respiration and lactate production. High flux in anabolic pathways imposed a strain on NADH homeostasis, which coincided with engagement of the proline cycle for mitochondrial redox shuttling. With acquisition of effector functions, cells increasingly relied on glycolysis as opposed to oxidative phosphorylation, which was, however, not linked to changes in mitochondrial abundance. In exhaustion, decreased effector function concurred with a reduction in mitochondrial metabolism, glycolysis and amino acid import, and an upregulation of quiescence-associated genes, TXNIP and KLF2, and the T cell suppressive metabolites succinate and itaconate.

**Discussion:**

Overall, these results identify multiple metabolic features that regulate quiescence, proliferation and effector function, but also exhaustion of CD8+ T cells during differentiation. Thus, targeting these metabolic checkpoints may be a promising therapeutic strategy for both prevention of exhaustion and promotion of stemness of anti-tumor T cells.

## Graphical Abstract

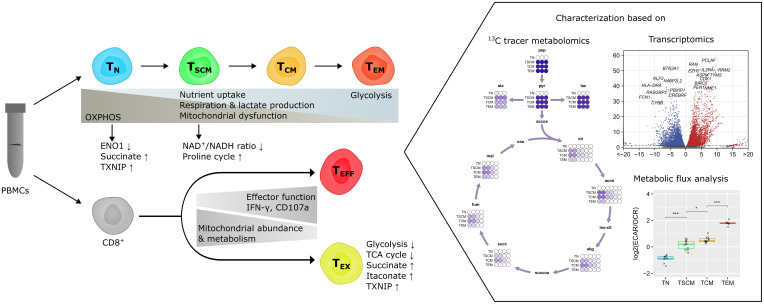

## Introduction

CD8^+^ T lymphocytes are cells of the adaptive immune system capable of acquiring cytotoxic functions. Following their development in the thymus, they persist in a quiescent state as naïve T cells until activation by cognate antigen. This results in the selective expansion of antigen-specific cells, which culminates in the mounting of an effective immune response. Due to their potentially disease-causing effects, cytotoxic cells require tight regulation and successful immune responses need to be rapidly terminated. Only a subset of antigen-specific cells survives long-term as memory cells for preparedness to antigen reencounter. Early studies revealed the presence of two major subpopulations of memory cells, central memory (T_CM_) cells that are primarily homed to lymph nodes and effector memory (T_EM_) cells with increased cytotoxic potential in the peripheral circulation (1). A rare population of stem cell memory (T_SCM_) cells has later been identified in mice (2) and humans (3). These cells are capable of long-term persistence, generate memory responses to antigen reencounter and produce T_CM_ and T_EM_ offspring. Further lineage analyses *in vivo* confirmed that stem-like T cells also sustain the primary immune response against cancer (4), viral infections (5), vaccines (6) and auto-immune targets (7). Activated, proliferating stem-like cells in the early phases of an immune response may differ in some aspects from preferentially resting stem-like cells during memory phases after antigen clearance, and stem-like CD8^+^ T cells have been described as both proliferative (8) and quiescent (9). While being functionally similar in the early and late phases of an immune response (5), they can transiently express activation and effector markers (10). Thus, although dedifferentiation of effector into memory cells may occur in some settings (11), the majority of studies supports a progressive “memory-first” differentiation model, where T_SCM_ cells with long-term persistence generate more differentiated, short-lived progeny for effector responses. This differentiation process needs to be tightly regulated to control the magnitude, effectiveness and dynamics of an immune response, and various regulatory points evolved for this purpose (12). For example, the quiescence of naïve T cells is enhanced by checkpoints such as VISTA (13), NRP1 restricts memory formation (14) and activation and effector functions are restrained by PD1 ligation (15).

Conceivably, the biosynthetic and bioenergetic demands of distinct T cell subsets are reflected in their metabolic needs. T cell activation induces a switch to glycolysis (16) to provide precursors for growth (17), whereas resting naïve and memory cells mainly rely on fatty acid oxidation (FAO) and oxidative phosphorylation (OXPHOS) to meet their basal energy requirements (18). Mitochondrial function has also been found to be impaired in and contribute to exhaustion (19). These metabolic programs are sometimes connected to specific immunometabolites that exert signaling functions, such as phosphoenolpyruvate (20), asparagine (21), kynurenine (22), succinate (23) or itaconate (24). In consequence, metabolic cues in the microenvironment have the potential to modulate T cell differentiation and may be co-opted not only by cancer cells, but also for cancer therapy.

Of particular interest in anti-cancer immunity are checkpoints that impair cytotoxicity in exhausted T cells. Exhaustion supposedly functions as a physiological adaptation to persistent antigen presence, but becomes a dysfunctional adaptation when tumor-associated microenvironmental signals result in reinforced immune suppression and subsequent failure to eradicate a tumor (25). Metabolic dysregulation associated with exhaustion is characterized by decreased glycolysis, OXPHOS and ATP production, whereas oxidative stress and ROS levels are elevated. Overall, this results in a disability of exhausted T cells to accomplish their role as cytotoxic cells (26). Checkpoint blockade is employed to overcome exhaustion in cancer therapies, but its success depends on the presence of stem-like CD8^+^ T cells (27). Of note, stemness of CAR-T cells has been shown to promote durable anti-tumor responses even at low doses with reduced side effects and lead to improved clinical outcomes (28). These less differentiated, stem-like CAR-T cells also exhibit enhanced *in vivo* expansion in patients and therefore allow for a decrease in infusion numbers, decreased *ex vivo* expansion and shorter manufacturing times. Thus, stemness and exhaustion are critical determinants of tumor-directed CD8^+^ T cell immunity. In-depth characterization of the metabolic profiles for various T cell subsets is hence of utmost importance for the development of novel therapeutic strategies in which T cells are forced to adopt a stem-cell memory phenotype to improve both immune checkpoint blockade as well as CAR-T cell therapy (29).

Several recent publications addressed CD8^+^ T cell metabolism during differentiation. Stable-isotope tracing has, for example, been employed by Hermans et al. (30), Wu et al. (21), or Ma et al. (31), who investigated T cell activation *in vitro* and *in vivo*, finding that effector cells had a less glycolytic phenotype and decreased pyruvate carboxylase (PC) flux *in vivo*. Moreover, single-cell approaches have shed light on subset heterogeneity. Levine et al. (32) used mass cytometry to demonstrate the emergence of a transient cluster of cells with high expression of proteins involved in glycolysis and OXPHOS. Fernández-García et al. (33) generated single-cell RNA sequencing data of *in vitro* activated CD8^+^ T cells to find an upregulation of both glycolysis and OXPHOS in early T cell clusters, followed by a shift towards glycolysis at later differentiation stages. However, stable-isotope tracing has not been used to investigate the whole spectrum of human CD8^+^ T cell subsets. Therefore, we used *in vitro* models of human CD8^+^ T cell activation and exhaustion to comprehensively profile the metabolic phenotypes associated with distinct differentiation stages under controlled conditions. Combined analysis of transcriptomics and ^13^C metabolomics data revealed metabolic checkpoints in glycolysis and the TCA cycle of quiescent naïve cells. Metabolic reprogramming towards aerobic glycolysis and anabolism coincided with engagement of the proline cycle for mitochondrial redox shuttling. Further differentiation towards effector cells was correlated with a progressive reliance on glycolysis. Moreover, glycolysis, mitochondrial metabolism and amino acid uptake were restricted in exhaustion. Overall, our findings highlight metabolic checkpoints in glycolysis and mitochondrial metabolism associated with quiescence, activation, effector differentiation and exhaustion of CD8^+^ T cells.

## Materials and methods

### Cell culture

Leukoreduction system chambers (34) from apheresis platelet donors were used as source of peripheral blood mononuclear cells (PBMCs) and were obtained from the Central Institute for Blood Transfusion and Immunology situated at the Tirol Kliniken GmbH in Innsbruck, Austria. At the time of the donation, platelet donors were in a healthy state and fulfilled the general requirements for donating blood in Austria. Leukoreduction system chambers from platelet donors were provided on informed consent after finishing the apheresis procedure. PBMCs were isolated by density gradient centrifugation on a Ficoll-plaque by using Lymphocyte Separation Medium (LSM-A) (Capricorn Scientific, Ebsdorfergrund, Germany). After Ficoll gradient separation, the PBMC fraction was collected and washed twice with ice-cold DPBS (Gibco/Thermo Fisher Scientific). PBMCs were counted under a hemocytometer and the viability was assessed *via* Trypan blue (Sigma Aldrich, St.Louis, USA). The cells were cultured in RPMI 1640 medium containing 10% Fetal Bovine Serum (FBS), 2 mM L-glutamine and 1% penicillin-streptomycin (all from Sigma Aldrich, St.Louis, USA) and incubated overnight at 37°C and 5% CO_2_ before starting the CD8^+^ T cell differentiation.

### Cell sorting

Naïve CD8^+^ T cells were sorted using a FACSAria I Sorter (BD Biosciences) by staining the isolated PBMCs with anti-CD8 FITC (BD Biosciences), anti-CD197 (CCR7) PE, anti-CD95 APC (both from BioLegend), anti-CD45RO PE-Cy7, anti-CD45RA BV421 and 7-Amino-Actinomycin D (7-AAD) (all from BD Biosciences). PBMCs were sorted into naïve T cells (T_N_, CD8^+^ CD197^+^ CD95^-^ CD45RO^-^ CD45RA^+^), collected into RPMI 1640 medium containing 1% FBS and counted manually before starting the differentiation experiments.

### *In vitro* differentiation model

For *in vitro* differentiation, naïve CD8^+^ T cells (1.5 x 10^6^ cells/mL and 1 x 10^6^ cells/mL) were distributed into four wells of a 24-well plate and activated for 8 days by adding prewashed anti-CD3/CD28 Dynabeads at a 1:1 bead-to-cell ratio (Invitrogen/Thermo Fisher Scientific, Massachusetts, USA) and human rIL-2 (30 U/mL) (Roche/Merck, Darmstadt, Germany) to the culture. To generate effector memory T cells, 1 x 10^6^ cells/mL were restimulated on day 7 by adding prewashed anti-CD3/CD28 Dynabeads at a 1:1 bead-to-cell ratio and human rIL-2 (30 U/mL) a second time to the culture. The cells were cultured in RPMI 1640 medium without glucose (Gibco/Life Technologies, Carlsbad, USA) where 11 mM D-glucose was added to 10% FBS, 2 mM L-glutamine and 1% penicillin-streptomycin (all from Sigma Aldrich, St.Louis, USA). 24 h before collection, culture medium was replaced with RPMI 1640 medium containing 11 mM of ^13^C labeled-glucose (Cambridge Isotope Laboratories, Massachusetts, USA). At indicated time points, naïve T cells (T_N_, day 1), stem cell memory T cells (T_SCM_, day 2), central memory T cells (T_CM_, day 5) and effector memory T cells (T_EM_, day 8) were collected and anti-CD3/CD28 beads were removed by using a DynaMag-2 magnet (Thermo Fisher Scientific, Massachusetts, USA) before further processing for flow cytometry, RNA sequencing and metabolomics analyses.

### Flow cytometry

To analyze cell surface marker during differentiation, 1 x 10^5^ PBMCs were labeled at every time point (day 1, 2, 5 and 8) with the same panel of human antibodies as used for cell sorting and analyzed by flow cytometry on a FACS Fortessa (BD Biosciences). UltraComp eBeads (Invitrogen/Thermo Fisher Scientific, Massachusetts, USA) were used for the compensation setup. The cell type fractions were determined as the following: Naïve T cells (T_N_, CD8^+^ CD197^+^ CD95^-^ CD45RO^-^ CD45RA^+^), stem cell memory T cells (T_SCM_, CD8^+^ CD197^+^ CD95^+^ CD45RO^-^ CD45RA^+^), central memory T cells (T_CM_, CD8^+^ CD197^+^ CD95^+^ CD45RO^+^ CD45RA^-^) and effector memory T cells (T_EM_, CD8^+^ CD197^-^ CD95^+^ CD45RO^+^ CD45RA^-^). Analysis was performed using FlowJo software.

### *In vitro* exhaustion model

*In vitro* exhausted CD8^+^ T cells were generated as previously described (35). Briefly, CD8^+^ T cells isolated from healthy donor PBMCs were stimulated by a 1:1 ratio of anti-CD3/28 activation beads (Miltenyi) in complete human T cell medium (RPMI1640 from Sigma containing 2 mM glutamine with the addition of 1 mM pyruvate, 1% penicillin-streptomycin, 10% heat-inactivated AB+ male human serum, 50 nM beta-mercaptoethanol in presence of 150 U/mL rhIL2 (Proleukin). The day after stimulation, cells were transduced with VSVg-pseudotyped Lentivirus encoding a human T cell receptor specific for NY-ESO-1 (gift from Natalie Rufer and Michael Hebeisen) (36). To generate exhausted T cells (T_EX_), TCR^+^ cells were stimulated every 3 days for four cycles with the HLA-A2^+^ T2 tumor cell line loaded with 1000 nM NY-ESO-1 SLLMWIQV peptide at a 1:3 effector to target ratio in T cell medium with 50 U/mL IL-2. For functional effector cells (T_EFF_), the T cells were expanded in the same media for 9 days before a single stimulation with the peptide loaded T2 cells as described above. 3 days post last stimulation, the T cells were sorted for live TCR^+^ CD8^+^ CD19^-^ CD14^-^ CD56^-^ cells and resuspended in RPMI 1640 medium containing 5 mM of ^13^C labeled-glucose at 1x10^6^ cells/mL with the addition of 50 U/mL IL-2. Cells were cultured in a 24-well plate for 24 h at 37°C with 5% CO_2_ before further processing for RNA sequencing and metabolomics analyses.

### Seahorse assay

Naïve T cells (T_N_), stem cell memory T cells (T_SCM_), central memory T cells (T_CM_) and effector memory T cells (T_EM_) were collected, counted, and centrifuged at 1500 rpm for 10 min at room temperature. Cells were resuspended at a concentration of 8x10^6^ cells/mL in Seahorse XF Base Medium (Agilent Technologies, Santa Clara, CA) supplemented with 1 mM pyruvate, 2 mM glutamine, 10 mM glucose (all from Sigma Aldrich, St.Louis, USA), and pH was adjusted to 7.4. Seahorse XFp Cell Culture Miniplates (Agilent Technologies, Santa Clara, CA) were precoated with poly-L-lysine (Sigma) and 1x10^5^ to 4x10^5^ cells were plated into each well. Miniplates were centrifuged at 300 g for 1 min with low-brake deceleration. Seahorse XFp Cell Energy Phenotype Tests (Agilent Technologies, Santa Clara, CA) were performed according to the manufacturer’s protocol on an XFp instrument and data analysis was performed using the Seahorse XFe Wave software.

### Metabolomics sample preparation

After 24 h culturing in ^13^C-containing medium, 1x10^6^ cells were collected at indicated time points and centrifuged at 2000 rpm for 1 to 3 min at 4°C. For medium extraction, 10 µL of the supernatant was added to 990 µL 80% methanol containing 2 µM of d27 myristic acid (pre-chilled to -80°C) (extraction buffer provided by the VIB Metabolomics Expertise Center). For cellular extraction, the supernatant was removed, the pellet was carefully washed with 1 mL of ice-cold 0.9% NaCl (Sigma Aldrich, St. Louis, USA) and this solution was then aspirated. Pellet was resuspended in 250 µL (T_EFF_ and T_EX_) or 300 µL (T_N_, T_SCM_, T_CM_ and T_EM_) of 80% methanol containing 2 µM of d27 myristic acid (pre-chilled to -80°C) and pulse vortexed three times for five seconds. Samples were stored at -80°C for 24 h, centrifuged at 1500 rpm for 15 min at 4°C and 250 µL of metabolite-containing supernatants of each sample were sent for analysis.

### LC-MS metabolomics measurements

10 µL of each sample was loaded into a Dionex UltiMate 3000 LC System (Thermo Scientific Bremen, Germany) equipped with a C-18 column (Acquity UPLC -HSS T3 1. 8 µm; 2.1 x 150 mm, Waters) coupled to a Q Exactive Orbitrap mass spectrometer (Thermo Scientific) operating in negative ion mode. A step gradient was carried out using solvent A (10 mM TBA and 15 mM acetic acid) and solvent B (100% methanol). The gradient started with 5% of solvent B and 95% solvent A and remained at 5% B until 2 min post injection. A linear gradient to 37% B was carried out until 7 min and increased to 41% until 14 min. Between 14 and 26 min the gradient increased to 95% of B and remained at 95% B for 4 min. At 30 min the gradient returned to 5% B. The chromatography was stopped at 40 min. The flow was kept constant at 0.25 mL/min and the column was placed at 40°C throughout the analysis. The MS operated in full scan mode (m/z range: [70.0000-1050.0000]) using a spray voltage of 4.80 kV, capillary temperature of 300°C, sheath gas at 40.0, auxiliary gas at 10.0. The AGC target was set at 3.0E+006 using a resolution of 140000, with a maximum IT fill time of 512 ms. Data collection was performed using the Xcalibur software (Thermo Scientific). The data analyses were performed by integrating the peak areas (El-Maven - Polly - Elucidata) (37).

### Metabolomics data analysis

Quantification thresholds were defined for each batch from three blank measurements as two standard deviations above blank means. Isotopologue intensities below blank means were set to zero and isotopologue intensities above blank means but below quantification thresholds were corrected by a linear mapping between zero and the quantification threshold. Only isotopologues with at least two biological replicates above quantification thresholds were used for statistical testing. Correction for natural isotope abundance was applied with the IsoCorrectoR R package 1.16.0 (38) in high-resolution mode. Corrected data were normalized between isotopologues by mean metabolite abundance and between samples by size factors consisting of total isotopologue sums weighted by their inverse relative variances. Isotopologue abundances were further cleaned from sample groups with more than 1/3 of the respective values below quantification threshold. Metabolite abundances were calculated from the sums of isotopologue abundances and fractions of isotopologues relative to isotopologue sums were used as mass isotopomer distributions. For differential abundance analysis, metabolite abundances were assessed in a linear mixed model using the nlme R package 3.1-162 and isotopologue fractions were modeled with beta regression using the glmmTMB R package 1.1.7. Celltype was treated as fixed and donor as random effect. The multcomp R package 1.4-23 was used for statistical hypothesis testing of particular contrasts and the resulting p-values were FDR-adjusted. Statistical analyses of isotopologue abundances using a linear mixed model and of fractional metabolite labeling using beta regression yielded similar conclusions and were thus not further discussed.

### RNA sequencing and data analysis

Naïve T cells (T_N_), stem cell memory T cells (T_SCM_), central memory T cells (T_CM_), effector memory T cells (T_EM_), full effector function T cells (T_EFF_) and exhausted T cells (T_EX_) were collected, snap frozen and total RNA was isolated using the RNeasy Plus Mini Kit (Qiagen) or RNeasy Plus Micro Kit (Qiagen GmbH-Austria, Hilden, Germany), including DNAse treatment and following the manufacturer’s instructions. All Isolated total RNA samples were quality validated and submitted to library preparation following the Lexogen QuantSeq 3’mRNA protocol (Lexogen GmbH, Vienna, Austria). The resulting libraries were multiplexed and sequenced with Ion Proton technology and Ion Hi-Q chemistry (Ion Torrent, Thermo Fisher Scientific, Vienna, Austria). CUTADAPT 4.0 was used to trim sequencing reads of low-quality bases and poly-A tails with the options -q 20, -O 10, -e 0.15, -m 10 and the adapter sequence -a A100 (39). We used the nf-core rnaseq pipeline 3.6 (10.5281/zenodo.6327553) with the trimming step disabled for the alignment of reads with STAR 2.7.9a to the human genome (GRCh38) and for the quantification of reads with SALMON 1.5.2 (40). The correct pairing of samples was confirmed by grouping samples to donors of origin with NGSCheckMate 1.0.0 (41). Raw counts were imported into R 4.1.3 and DESeq2 1.38.3 was used with IHW 1.26.0 to test for differentially expressed genes (42). For analyses of biological functions, we compiled a collection of gene sets from gene ontology (GO), HALLMARK, KEGG and the metabolic databases MitoCarta 3.0 (43) and MetabolicAtlas 3.3 (44). For the differentiation samples (T_N_, T_SCM_, T_CM_ and T_EM_), significantly different genes with padj < 0.05 were partitioned into groups of up- (U) and downregulation (D) according to their z-scaled expression values. The resulting gene clusters were further used for gene set overrepresentation analysis (ORA) with the ClusterProfiler R package 4.6.2 (45). For samples from the exhaustion experiments, we performed gene set enrichment analysis (GSEA) with ClusterProfiler on genes ranked by the Wald test statistic from DESeq2. Raw bulk RNA sequencing data were retrieved from the GEO database for accession numbers GSE121226 (46), GSE140430 (47), GSE147398 (48) and GSE179609 (49) and uniformly processed. The single-cell CD8^+^ T cell atlas was downloaded from https://singlecell.mdanderson.org/TCM (50). We then identified the top expressed genes in each of our subsets by one-vs-all comparisons and calculated the enrichment scores of these signatures in the normalized bulk and pseudo-bulk reference samples using gene set variation analysis (51). The regression model for mitochondrial abundance prediction was based on public data from (52) and (53).

## Results

### Transcriptomic characterization of CD8^+^ T cell subsets reveals metabolic reprogramming and differential expression of metabolic regulators

**Figure 1:**
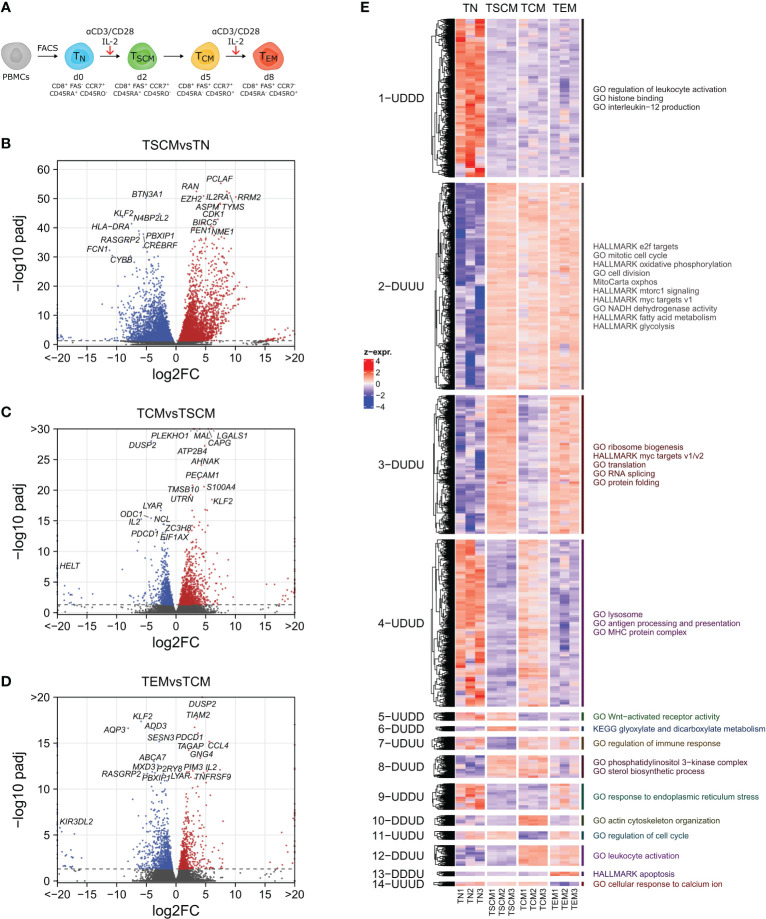
Transcriptomic characterization of CD8^+^ T cell subsets. **(A)** Experimental Setup. Sorted naïve CD8^+^ T cells (T_N_) are stimulated with anti-CD3/anti-CD28 beads in presence of IL-2. At day 2, a population of FAS^+^ stem cell memory cells (T_SCM_) is established that further differentiates into a population of central memory (T_CM_) cells that peaks at or after day 5, coinciding with an isoform switch in CD45. Additional restimulation induces terminal differentiation and downregulation of the lymph-node homing receptor CCR7. **(B-D)** Volcano plots showing differentially expressed genes (padj < 0.05) between consecutively sampled subsets. **(E)** Heatmap of differentially expressed genes partitioned into patterns of up- and downregulation during differentiation (D=down, U=up), and pathway overrepresentation analysis of genes in selected clusters.

To study the differentiation dynamics of human T cells, we employed an *in vitro* model of naïve T cell (T_N_) activation (**Figure 1A**). Sorted CD8^+^ T_N_ cells from three healthy donors were stimulated with anti-CD3/CD28 beads and IL-2 to drive activation and differentiation towards stem-like (T_SCM_) and central memory (T_CM_) cells, followed by restimulation to promote full differentiation of effector (T_EM_) cells. Based on the expression of a panel of markers, we confirmed the presence of these distinct phenotypes at different time points by flow cytometry (**Figures S1A-C**), showing that our data are consistent with a differentiation model where the majority of activated cells acquire an effector phenotype after passing through a stem-like progenitor phase, followed by a contraction phase at the end.

Based on our initial observation of surface marker dynamics during differentiation, we characterized gene expression changes on a global scale by RNA sequencing, followed by differential gene expression analysis. We compiled a collection of publicly available RNA sequencing datasets that include T_SCM_ or T_CM_ samples to demonstrate the similarity of our samples with these T cell subtypes (**Figure S2A**). In addition, we compared our subsets to a recently published single-cell atlas of tumor-infiltrating CD8^+^ T cells to confirm their relevance *in vivo* (**Figure S2B**). **Figure S2C** shows the expression levels of selected marker genes. Quiescence- (*FOXO1*, *BTG1/2*) and stemness-associated genes (*TCF7*) were highly expressed in naïve cells. Activation markers (*ILRA*, *CD27*, *CD28*) were upregulated following activation and effector proteins (*IFNG*, *GZMB*) were most strongly upregulated in T_EM_ cells. Among inhibitory receptors, *VSIR* (VISTA) was highly expressed in T_N_, *PDCD1* was transiently upregulated following the initial stimulation, and both *PDCD1* and *LAG3* were upregulated following re-stimulation in T_EM_ cells.

Differential gene expression analysis revealed global transcriptomic changes between these CD8^+^ T cell subsets (**Table S1**), with the strongest effect following the initial stimulation of T_N_ cells. At a significance level of 0.05, 7076 genes were differentially expressed between T_N_ and T_SCM_ cells (**Figure 1B**), 2164 genes changed between the T_SCM_ and the T_CM_ subset (**Figure 1C**), and 1591 differed significantly upon restimulation and further development of T_CM_ into T_EM_ cells (**Figure 1D**). *KLF2*, which has been previously associated with quiescence (54), was highly expressed in T_N_ and downregulated upon activation in T_SCM_ cells. Besides classical activation markers, genes involved in cell cycle regulation (*CDK1*, *AURKB*) and nucleotide biosynthesis (*TYMS*, *RRM2*) were among the highest upregulated genes in T_SCM_, along with other key differentially expressed metabolic genes like *GAPDH*, *ENO1*, *FABP5*, *LDHA* or *TXNIP*. Compared to T_SCM_ cells, T_CM_ cells upregulated several genes also found in T_N_ cells (*KLF2*, *SLFN5*) and downregulated several metabolic genes (*ODC1*, *NAMPT*). T_EM_ cells had high expression of genes associated with effector cells, including *PDCD1*, *IL2*, *FASLG* and *IFNG*.

As many differentially expressed genes were shared between subsets, we partitioned these genes into clusters of up (U)- and downregulation (D) and performed gene set overrepresentation analysis on the individual clusters (**Figure 1E**, **Table S2**). Gene sets overrepresented only in T_N_ cells (cluster 1, 1-UDDD) included pathways involved in histone binding and modification, transcriptional repression and in the generic activation of immune cells. Various processes related to cell cycle control, DNA replication and cell proliferation, as well as MYC, mTORC1 and E2F signaling were highly enriched in all activated subsets (2-DUUU). This concurred with an upregulation of metabolic processes including OXPHOS, TCA cycle, mitochondria, glycolysis, fatty acid metabolism, nucleotide biosynthesis, NADH metabolism and succinate dehydrogenase activity, indicating major metabolic reprogramming due to increased enzyme expression. Two clusters were mainly comprised of transiently activated genes that depended on acute (re-) stimulation (i.e., genes highly expressed in T_SCM_ and T_EM_ cells). Therein, MYC signaling, ribosome biogenesis and metabolic pathways were even further upregulated (3-DUDU), whereas lysosomal pathways and antigen processing were downregulated (4-UDUD). Wnt signaling was a key feature of T_N_ and T_SCM_ cells (5-UUDD). Specifically enriched in T_SCM_ (6-DUDD) was “KEGG glyoxylate and dicarboxylate metabolism”, and downregulated genes (7-UDUU) were involved in leukocyte cell-cell adhesion, apoptosis and immune activation and differentiation. Genes shared by T_SCM_ and T_CM_ were involved in PI3K signaling and amino acid transport (8-DUUD). Actin cytoskeleton remodeling and leukocyte migration were characteristically activated in T_CM_ cells (10-DDUD), whereas cell cycle, Wnt signaling and histone acetylation were repressed (11-UUDU). Genes up in the T_CM_ and T_EM_ subsets (12-DDUU) included DNA deamination (nucleotide salvage), IFN-γ response, secretion and leukocyte degranulation. Apoptosis was most strongly upregulated in T_EM_ cells (13-DDDU), together with hypoxia and lymphocyte migration, while they were devoid of Ca^2+^ signaling (14-UUUD). The top pathways specifically upregulated in each subset are summarized in **Figure S2D**.

Thus, these results show that *in vitro* differentiation reflects the main processes of *in vivo* immune responses (activation, proliferation, migration, contraction) and recovered their main regulatory cascades, including Wnt and Ca^2+^ signaling. Several pathways that orchestrate metabolic reprogramming during T cell activation and differentiation, such as mTOR and MYC, and key metabolic genes (*ENO1*, *TYMS, SDHA, SDHB, SDHC*) were upregulated following T cell activation, whereas the levels of the metabolic regulator *TXNIP* decreased.

### ^13^C tracer analysis reveals ENO1 as quiescence checkpoint in T_N_ cells

**Figure 2:**
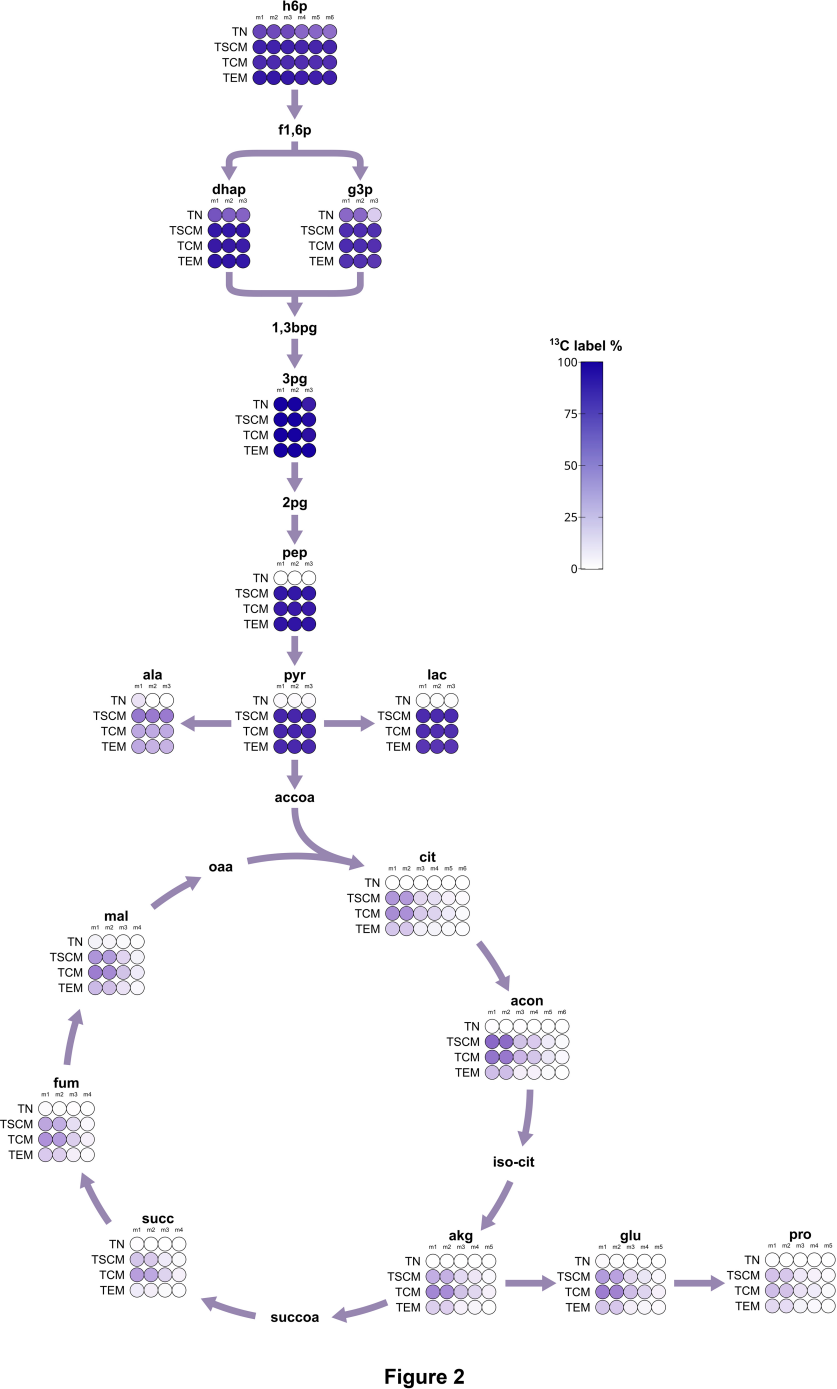
^13^C flux map of central carbon metabolism. Flux map showing the ^13^C labeling as the cumulative isotopologue distribution of selected metabolites. Color intensity indicates the fraction of labeled carbon atoms per metabolite.

As the transcriptomic analyses indicated prominent changes in cellular metabolism, we went on to directly investigate metabolic pathway activities by performing ^13^C tracing experiments followed by LC-MS measurements. First, we generated a global qualitative map of tracer fluxes in central carbon metabolism (**Figure 2**). U-^13^C-glucose was readily imported and shunted into glycolysis within 24 h of labeling in all subsets, including naïve T cells, resulting in full ^13^C labeling of metabolites in upper glycolysis. In T_N_ cells, however, no labeling was detected downstream of phosphoglycerate (3pg), indicating that no glycolytic flux went into the production of phosphoenolpyruvate (pep). This was in line with a pronounced transcriptomic downregulation of *ENO1* mRNA in T_N_ cells, which we assumed to be the major driver of the enolase reaction, as *ENO2* was only very lowly expressed (**Figure S3**). Enzymes of the rate-limiting steps of glycolysis were also differentially expressed (*HK1*, *PFK*, *PK*). Whereas *PKLR* was lowly expressed in T_N_ cells and even further downregulated upon activation, *PKM* was abundant and increased further after activation, in line with its known role to promote anabolism by increasing the availability of glycolytic intermediates (55). Thus, T_N_ cells had a pronounced suppression of glycolytic flux and did not oxidize glucose in the TCA cycle, indicating that these cells used carbon sources other than glucose for OXPHOS. In contrast, activated cells showed a consistent pattern of m+2 incorporation into the TCA cycle, indicating that glycolytic flux was directed there primarily *via* acetyl-CoA. To a lesser extent, m+3 labeling was observable at a mean m+3/m+2 ratio of 0.15, which likely originates from anaplerotic flux *via* pyruvate carboxylase (PC), and m+4 isotopomers originating from the condensation of m+2-labeled oxaloacetate with m+2-labeled acetyl-CoA in higher-order turns of the cycle. High flux through the PC reaction has previously been described as a characteristic of *in vitro* activated T cells with an m+3/m+2 ratio well above 0.5, whereas *in vivo* ^13^C tracing showed a predominance of m+2 labeling in the TCA cycle (31). Thus, our subsets exhibit an m+3/m+2 ratio similar to that observed *in vivo*, in line with low *PC* expression.

### ^13^C metabolomics identifies proline cycle activity in T_SCM_ cells

**Figure 3:**
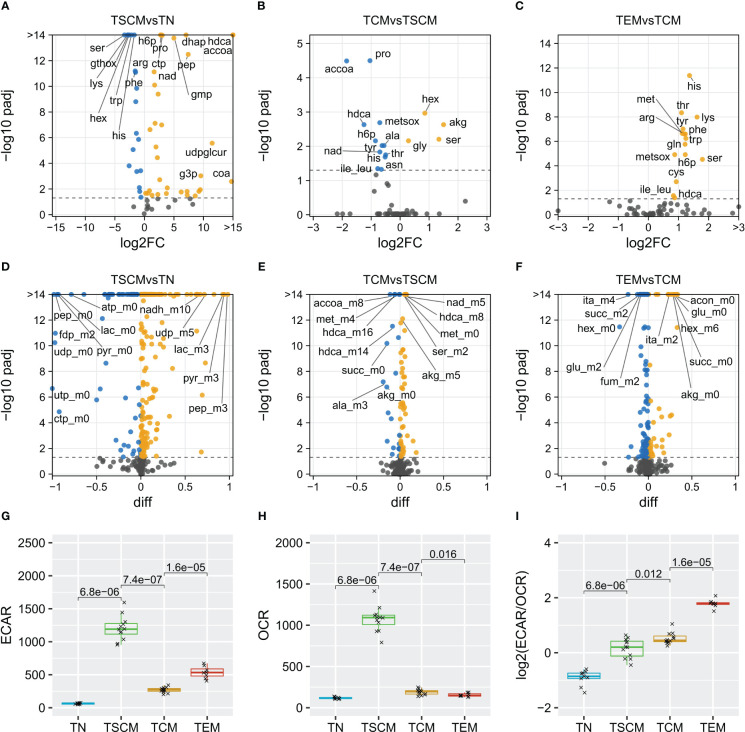
Metabolic characterization of CD8+ T cell subsets. Volcano plots of metabolite abundances **(A–C) ** and isotopomer fractions **(D–F)**. Rates of **(G)** extracellular acidification (ECAR, mpH min-1 10-6 cells-1) and **(H)** oxygen consumption (OCR, pmol O2 min-1 10-6 cells-1) measured on a Seahorse Analyzer, plotted as log-ratios **(I)** and tested for differences using the Wilcoxon test.

To study the metabolic dynamics of CD8^+^ T cells during activation in more detail, we analyzed the LC-MS data to detect significant differences in both metabolite abundances (**Figures 3A–C**; **Table S3**) and labeling states (**Figures 3D–F**; **Table S3**). Compared to T_N_, T_SCM_ cells had decreased intracellular levels of hexoses (hex), but increased levels of hexose-6-phosphates (h6p), glycolytic intermediates (dhap, g3p) and lactate (lac) (**Figure 3A**). Analysis of mass isotopomer distributions further revealed significantly increased labeling of glycolytic products, in particular, a shift from unlabelled to fully labeled pyruvate (pyr_m3) and lactate (lac_m3), and to 50% labeled alanine (ala_m3) (**Figure 3D**). As described above, the expression of glycolytic genes was increased. Thus, the suppression of glycolytic flux in naïve T cells was released upon activation. Metabolites in the beginning of the TCA cycle had 30% increased labeling derived from acetyl-CoA (citrate-m+2, aconitate-m+2), which declined to 10-20% further downstream. Surprisingly among the TCA cycle metabolites, succinate levels were increased and the fumarate/succinate ratio was decreased in T_N_ cells (**Figure S4A**). Expression of succinate, fumarate and malate dehydrogenases was also decreased (**Figure S5**), implying substrate accumulation due to downregulation of succinate dehydrogenase (SDH) activity in T_N_ cells. As expected, T_SCM_ cells also upregulated several biosynthetic pathways. Aspartate, glutamate and proline exhibited the same labeling patterns as their respective precursors in the TCA cycle due to ongoing biosynthetic flux. Nucleotide, glycosylation (UDP-GlcNAc) and glucuronidation (UDP-glucuronate) substrate levels and labeling were increased and the glycine/serine ratio was higher (**Figures S4B, C**), indicating increased serine biosynthesis and demand for 1C units.

The GSSG/GSH (oxidized and reduced glutathione, respectively) ratio was higher in T_N_ cells compared to all other subsets (**Figure S4C**). Moreover, in T_SCM_ cells, most amino acid levels were decreased, whereas biosynthesis and the expression of amino acid transporters (*SLC7A5*, *SLC3A2*, *SLC1A5*, *SLC38A5*) were simultaneously upregulated. Notable exceptions among amino acids were significantly higher concentrations of proline and alanine in T_SCM_ compared to T_N_ cells. T_SCM_ cells also had the highest NADH/NAD^+^ ratio and increased labeling of NADH. Continuous regeneration of NAD^+^ from NADH is required to support high rates of glycolytic flux, which can be achieved in the LDH reaction together with conversion of pyruvate to lactate. As expected, we found increased *LDHA* expression (log2FC=2.7). However, we also found production of alanine from pyruvate, as well as increased flux into the TCA cycle, and both of these pathways can not be directly used to regain NAD^+^. Rather, catabolisation of pyruvate and subsequent oxidation of TCA cycle intermediates in mitochondria produces additional NADH from NAD^+^. NADH is eventually oxidized to NAD^+^ in OXPHOS, but then needs to be transferred across the mitochondrial membranes to be used in glycolysis. Several shuttle systems exist for this purpose. We found increased expression of genes involved in the malate-aspartate shuttle and partial upregulations of the glycerol-3-phosphate shuttle and the citrate-malate shuttle (**Figure S6**). In addition, genes involved in the proline cycle were upregulated (*PYCR1*, *PYCR2* and *ALDH18A1*). As we also found an increase in proline levels and labeling, we therefore suggest that T_SCM_ cells utilize the proline cycle to maintain NADH balance. In T_CM_ cells, proline levels were moderately reduced compared to T_SCM_ cells (**Figure 3B**), *PYCR1* was downregulated and the glycolytic intermediate dihydroxyacetone phosphate (dhap) was decreased. Labeling of TCA cycle metabolites was higher in T_CM_ cells, most likely due to a decrease in both, draining cataplerotic reactions and replenishing anaplerosis (**Figure 3E**). Decreased glutamine catabolization is in line with a lower activity of MYC, a known driver of glutaminolysis. Compared to T_CM_ cells, the intracellular levels of most amino acids were increased in the T_EM_ subset (**Figure 3C**), reflecting decreased consumption, as the expression of importers was only marginally increased or even decreased (**Figure S7**). Whereas glycolytic intermediates were increased and the labeling remained high in glycolysis, the labeled fractions of TCA cycle intermediates were decreased (**Figure 3F**). Thus, high glycolytic flux, decreased biosyntheses and decreased entry of glucose carbon into the TCA cycle were characteristic of T_EM_ metabolism.

In summary, the ^13^C tracing data revealed a quiescence-associated metabolism in T_N_ cells. Reprogramming to an anabolism-oriented phenotype was indicated by high rates of glycolysis and nucleotide biosynthesis in T_SCM_ cells, which also coincided with an upregulation of the proline cycle. T_CM_ metabolism was qualitatively similar to the T_SCM_ phenotype, but compatible with lower rates in biosynthetic pathways. A further increase in the reliance on glycolysis with a concomitant decrease in other pathways was the main metabolic feature of the T_EM_ subset.

### Extracellular flux analysis reveals a shift to glycolysis during effector differentiation

We then performed extracellular flux analysis on a Seahorse Analyzer to relate the observed intracellular metabolic fluxes to extracellular acidification rates (ECAR) and oxygen consumption rates (OCR) (**Figures 3G, H**). Unstimulated T_N_ cells had only basal ECAR and OCR. T_SCM_ cells were the most active subset and operated at maximum capacities of both ECAR and OCR. Both T_CM_ and T_EM_ cells had low OCRs that were only slightly higher than that of T_N_ cells, but had higher spare respiratory capacity. ECAR was similarly decreased in T_CM_ cells, but upon restimulation and effector differentiation, ECAR significantly increased to rates approximately half of those of T_SCM_ cells in the T_EM_ cells. Despite these pronounced differences in metabolic rates between subsets, we found a progressive and gradual increase in the ECAR/OCR ratios in consecutive subsets (**Figure 3I**), suggesting that CD8^+^ T cells increasingly rely on glycolysis during differentiation. To further investigate these findings on a transcriptional level, we checked the expression of OXPHOS subunits (**Figure S8**). Unexpectedly, expression of most of the respiratory complexes was not decreased or even increased in T_EM_ cells. Additionally, a regression model trained on public datasets with mitochondrial copy numbers available predicted a high abundance of mitochondria in T_EM_ cells (**Figure S9A**), indicating that the observed shift towards glycolysis was not due to decreased mitochondrial abundance or changes in mitochondrial gene expression.

### Exhausted T cells are metabolically impaired and share features of quiescent subsets

**Figure 4:**
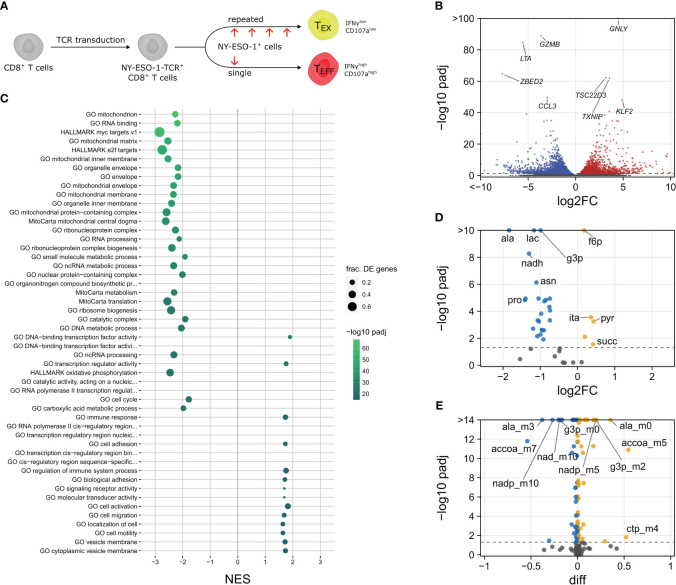
Characterization of exhausted CD8^+^ T cells. **(A)** Experimental Setup. **(B)** Volcano plot of differentially expressed genes. **(C)** Normalized enrichment scores (N_ES_; N_ES_ > 0 up in T_EX_ vs. T_EFF_, N_ES_ < 0 down in T_EX_ vs. T_EFF_) and fractions of differentially expressed genes per set (frac. DE genes) of selected significantly enriched gene sets. Volcano plots of differentially abundant metabolites **(D)** and isotopologues **(E)**.

Finally, we investigated whether our observations were related not only to the differentiation but also to the proper function and dysfunction of T cells. We adopted an *in vitro* exhaustion model (35) to generate control effector and hypofunctional exhausted T cells (**Figure 4A**). Briefly, CD8^+^ T cells from healthy human donors were transduced with a T cell receptor specific for the NY-ESO-1 cancer antigen, and the transgenic T cells were stimulated with NY-ESO-1 either acutely or repeatedly to induce full effector function (T_EFF_) or a hypofunctional state of exhaustion (T_EX_), respectively. Analysis of the RNA sequencing data showed 3625 differentially expressed genes between T_EX_ and T_EFF_ cells (**Figure 4B**; **Table S1**). Whereas most effector molecules (*GZMB*, *LTA*) were decreased, expression of *GNLY* was increased in exhausted cells. As in quiescent naïve T cells, the transcription factors *KLF2*, *TCF7* and *BTG1* were upregulated. Notably, among the genes with metabolic functions, *TXNIP* had the most differentially upregulated transcript in T_EX_ cells. We further confirmed differential regulation of these genes (**Table S4**) in a previously published dataset (35).

Gene set enrichment analysis (**Figure 4C**; **Table S5**) found an upregulation of DNA-binding transcription factor activity, with *KLF2* and *TCF7* as the top contributing genes. Also upregulated were cell adhesion terms and “GO negative regulation of leukocyte mediated cytotoxicity”. A downregulation was observed for gene sets involved in the main signaling pathways involved in T cell activation, including MYC, E2F, mTORC1 signatures. Moreover, terms related to cell cycle, ribosomes, OXPHOS, glycolysis and amino acid metabolism were downregulated. Most striking, however, was the depletion of mitochondrial components. Consistently, the predicted mitochondrial abundance was significantly decreased in T_EX_ cells (**Figure S9B**).

We further performed ^13^C tracer studies with exhausted and control effector cells (**Table S6**). Whereas pyruvate levels were slightly higher in T_EX_ compared to T_EFF_ cells, lactate levels were decreased and less labeled (**Figures 4D, E**). Consistent with lower glycolytic flux, *LDHA* and key glycolytic enzymes were expressed at lower levels in T_EX_ cells. Most amino acids had decreased intracellular concentrations, particularly alanine and proline. Genes of the proline cycle were strongly downregulated (*PYCR1*, *PYCR2*, *PYCR3* and *ALDH18A1*) while the NAD^+^/NADH ratio remained high, suggesting that T_EX_ cells could easily maintain redox balance even under low lactate production. Alanine production is usually coupled to glycolytic flux and its labeling was likewise decreased. Presumably, T_EX_ cells need less nutrients for protein synthesis and growth, so lower amino acid levels would hint towards decreased uptake. Indeed, we found that several of the main amino acid transporters were downregulated in T_EX_ cells, including *SLC7A5*, *SLC1A5* and *SLC38A5* (**Figure S7**). In the TCA cycle of T_EX_ cells, succinate was slightly but significantly increased and the fumarate/succinate ratio was decreased (**Figure S4F**), indicating lower SDH activity. Expression of SDH was moderately decreased, as well as MDH2 and, most pronouncedly, FH. In addition, the TCA-cycle-derived itaconate showed a small but significant increase in T_EX_ cells.

Together, these data indicate that exhausted cells had reduced effector functions and partially activated signaling pathways associated with quiescent cells. T_EX_ cells were less metabolically active than T_EFF_ cells, had lower glycolytic flux and were depleted in intracellular amino acids due to restricted uptake. In addition, changes in the TCA cycle were associated with lower SDH activity, succinate and itaconate accumulation and mitochondrial abundance was decreased.

## Discussion

Metabolic reprogramming of T cells is known to shape the outcomes of immune responses to infections and cancer and is thus of great interest for clinical applications. In this study, we carried out comprehensive characterizations of the metabolic programs of human CD8^+^ T cell subsets to provide, for the first time, a direct comparison of ^13^C tracer fluxes in naïve, differentiating and exhausted CD8^+^ T cells. Our results show that the glycolytic enzyme *ENO1* was selectively downregulated in naïve T (T_N_) cells to suppress lower glycolysis, thereby constituting a novel quiescence checkpoint. Upon activation, stem cell memory (T_SCM_) cells operated at maximum metabolic capacity and maintained their metabolic fitness by engaging the proline cycle. Over the course of differentiation from T_N_ to T_SCM_, central memory (T_CM_) and effector memory (T_EM_) cells, CD8^+^ T cells underwent a progressive shift from OXPHOS towards glycolysis, independent of their baseline metabolic activities. Finally, exhausted cells (T_EX_) shared several metabolic similarities with quiescent T_N_ cells, such as decreased glycolysis, reduced TCA cycle flux and an upregulation of *TXNIP*. Moreover, we found an increase in succinate and itaconate abundance in T_EX_ cells.

Thus, our findings highlight several metabolic regulatory points of CD8^+^ T cell differentiation. Checkpoints in control of T_N_ exit from quiescence have been discussed previously (56). Here, we show that T_N_ cells displayed only basal metabolic rates and had flux constraints in lower glycolysis and the TCA cycle. The breakpoint in glycolysis was centered around enolase (ENO1) (**Figure 2**), which has a critical role in supplying phosphoenolpyruvate for T cell activation. Phosphoenolpyruvate inhibits SERCA activity by promoting cysteine oxidation, thereby prolonging TCR-induced Ca^2+^ signaling (20). Decreased phosphoenolpyruvate levels due to glucose deprivation have thus been shown to induce T cell anergy and tumor-infiltrating lymphocyte (TIL) dysfunction. Moreover, ENO1 activity was post-translationally impaired in mouse and human TILs, and this suppression was prevented by combinatorial checkpoint therapy (57). Thus, our findings suggest that ENO1 is selectively downregulated in naïve T cells to ensure suppression of activation and maintenance of quiescence. Basal TCA cycle activity in T_N_ cells was associated with low expression of enzymes of later stages of the TCA cycle, resulting in reduced flux through the SDH, FH and MDH reactions and an accumulation of succinate. SDH has been found to be necessary for T cell activation (58) and its downregulation may therefore represent another mechanism to ensure quiescence.

Upon activation, cell cycle entry and differentiation, T_SCM_ cells proliferate rapidly and turn up their biosynthetic machinery to operate at its maximum, consistent with our ECAR and OCR measurements (**Figure 3**). Limiting factors for biosynthesis and growth include protein synthesis, ATP production and NADH balance (17), all of which are upregulated in T_SCM_ cells. Besides lactate secretion, we also noticed export of alanine and pyruvate, as previously found in cancer cells (59) and T cells (60), respectively. In addition to high metabolic demands for NAD^+^, this may restrict the ability to regenerate NAD^+^ *via* lactate dehydrogenase. We further found that T_SCM_ cells had a low NAD^+^/NADH ratio (**Figure S4D**) and upregulated proline biosynthesis (**Figure 3**, **Figure S6**), possibly to engage the proline cycle to transfer reducing equivalents from the cytosol to mitochondrial OXPHOS and thereby regenerate cytosolic NAD^+^. Notably, overexpression of *PRODH2*, a gene of the proline cycle, has been found to enhance the metabolic fitness and anti-tumour efficacy of CAR-T cells in a mouse model (61) and high expression of *Prodh* and proline biosynthesis genes was found in clusters of 24-h-activated T cells in a recent single-cell RNA sequencing study (33). In addition, maintenance of a sufficiently high NAD^+^/NADH ratio by the malate-aspartate shuttle is needed for serine biosynthesis, 1C-metabolism and nucleotide biosynthesis in CD8^+^ T cells (60), processes we found to be particularly active in T_SCM_ cells. Thus, our results indicate that NADH balance is a limiting factor for T_SCM_ cell metabolism and several metabolic systems, including the proline cycle, are activated to increase the capacity for NAD^+^ regeneration.

A switch-like transition from OXPHOS to glycolysis has been consistently found in many studies of T cell metabolism (62). In line with recent publications (32) (33), we found an upregulation of both OXPHOS and glycolysis early after activation. Our ^13^C tracing and Seahorse data (**Figure 3**) extend these findings by revealing a simultaneous gradual, but progressive shift towards glycolysis during differentiation, which was independent of the baseline metabolic activities and proliferation rates of the cells. Rather, the fraction of glycolytic flux that was used in OXPHOS steadily decreased. Notably, the GAPDH protein can function as a sensor of glycolytic flux that, when not bound to substrate, suppresses translation of IFN-γ mRNA in CD8^+^ T cells (63), explaining the necessity of glycolytic flux in effector cells. High glycolytic flux provides intermediates for biosynthetic pathways, while a considerable fraction thereof is also used to produce lactate. In the extracellular environment, exported lactate may contribute to the acidification of lymph nodes (64), support VISTA signaling (65) and promote T cell stemness (66). Intracellularly, increased lactate levels may promote lactylation (67), a post-translational histone modification that preferentially affects metabolic enzyme expression (68). Moreover, extracellular lactate treatment has also been found to promote the stemness of CD8^+^ T cells by inhibiting histone deacetylation (69). Inhibition of LDH prevented effector differentiation and promoted stemness of CD8^+^ T cells, whereas transient inhibition allowed for full differentiation with an increased pool of stem-like cells (30).

The glycolytic shift may be directly explained by decreased mitochondrial abundance, however, transcriptomic signatures of mitochondria were not reduced in T_EM_ cells (**Figure S9A**). Asymmetric inheritance of mitochondria during T cell differentiation, though, can lead to an enrichment of dysfunctional mitochondria with decreased capabilities for OXPHOS in effector cells (70). Recent investigations further suggested that mitochondrial translation, rather than metabolism, is needed for the synthesis of selected effector proteins (71) and is enhanced during inflammation by fever (72). In contrast, exhausted cells exhibited a profound decrease in mitochondrial abundance (**Figure S9B**), suggesting that a T-cell-intrinsic exhaustion program that is independent of microenvironmental stresses is sufficient to induce mitochondrial insufficiency. Exhausted T cells also had decreased glycolytic flux (**Figure 4**), as previously described (73).

In addition to decreased glycolysis, T_EX_ cells shared multiple other features associated with quiescent T_N_ cells, such as an upregulation of *KLF2*, *TCF7, BTG1* and *TXNIP*, a downregulation of *SDHA* and an increase in succinate. TXNIP is an endogenous inhibitor of thioredoxin, a critical component of cellular redox regulation, and its repression by MYC enables thioredoxin-dependent DNA synthesis upon T cell activation (74). In addition, TXNIP also mediates glycolytic reprogramming of T cells during exit of quiescence (75) and can be downregulated by TCR signaling (76), by co-stimulatory signals (77) and *via* a glucose-sensing mechanism (78). To our knowledge, TXNIP has not been directly linked to T cell exhaustion and may contribute to the suppression of glycolysis, decreased ROS protection and mitochondrial dysfunction in T_EX_ cells. Uptake of succinate can suppress T cell effector functions (79), while another report suggested that cytotoxic T cells continuously need to produce and export succinate to promote autocrine signaling *via* SUCNR1 (23). The TCA-cycle-derived metabolite itaconate has been reported to inhibit SDH activity (80). Although we failed to detect significant differences in itaconate levels during differentiation, we found labeling patterns of itaconate that matched TCA cycle compounds in several T cell subsets, and an increase in itaconate abundance in T_EX_ cells (**Figure 4**). Whereas initial investigations did not find any effects of itaconate in CD8^+^ T cells (58), a more recent study reported suppressive effects of external itaconate supplementation, which lead to succinate accumulation and reduced proliferation (81). In addition, itaconate may also suppress glycolysis through post-translational mechanisms (82), consistent with our observation of decreased glycolysis in exhausted cells. These results therefore suggest that itaconate also has a T-cell-intrinsic role and may contribute to exhaustion by endogenous production.

In summary, our results highlight the landscape of intrinsic metabolic checkpoints of CD8^+^ T cell metabolism in various differentiation states. Modulation of metabolic programs by activating or inhibiting these targets, even if only transiently, has the potential to arrest differentiation or promote subset conversion. The feasibility of this approach has previously been demonstrated for mTOR (83) or Wnt inhibition (8), nutrient restriction (84) or lactate dehydrogenase inhibition (30). Thus, these results may provide onset points in various therapeutic settings, such as the *ex vivo* conditioning of T cells in cellular therapies for the treatment of cancers.

## Data availability statement

Code to reproduce the analyses is available at https://github.com/icbi-lab/kirchmair_2023 and functions for the analysis of ^13^C metabolomics data are available as an R package at at https://github.com/AlexanderKirchmair/c13ms. RNA sequencing data of samples from differentiation experiments are available at the Gene Expression Omnibus (GEO) database under accession number GSE234099 and samples from exhaustion experiments under accession number GSE234100. Processed metabolomics and Seahorse data tables are available on github.

## Ethics statement

The studies involving humans were approved by Central Institute for Blood Transfusion and Immunology, Tirol Kliniken GmbH. The studies were conducted in accordance with the local legislation and institutional requirements. Written informed consent for participation was provided from all platelet donors.

## Author contributions

ZT: Conceptualization, Funding acquisition, Supervision, Writing – review & editing. AK: Data curation, Formal Analysis, Software, Writing – original draft. NN: Investigation, Methodology, Writing – review & editing. GL: Investigation, Methodology, Writing – review & editing. MT: Investigation, Methodology, Writing – review & editing. AnK: Methodology, Writing – review & editing. AnS: Resources, Writing – review & editing. PH: Resources, Writing – review & editing. PS: Methodology, Writing – review & editing. SS: Writing – review & editing. AS: Methodology, Writing – review & editing. AZ: Methodology, Writing – review & editing. BG: Methodology, Writing – review & editing.

## Supplementary material

Supplementary Figure 1
**(A)** Flow Cytometry gating strategy to identify differentiation stages of naïve CD8^+^ T cells. Naive CD8^+^ T cells were stained and after doublet and dead cell removal by using forward scatter height (FSC-H) and area (FSC-A) and 7-AAD staining, CD8^+^ T cell population was further gated into different subsets during the course of 8 days of differentiation (exemplarily plots of Donor 1). Differentiation stages were defined based on the differential expression of CD95, CD45RA, CD45RO and CD197 on CD8^+^ T cells. T_N_ (naïve T cells, CD8^+^ CD197^+^ CD95^-^ CD45RO^-^ CD45RA^+^); T_SCM_ (stem cell memory T cells, CD8^+^ CD197^+^ CD95^+^ CD45RO^-^ CD45RA^+^); T_CM_ (central memory T cells, CD8^+^ CD197^+^ CD95^+^ CD45RO^+^ CD45RA^-^); T_EM_ (effector memory T cells, CD8^+^ CD197^-^ CD95^+^ CD45RO^+^ CD45RA^-^). **(B)** Differentiation dynamics. Changes in subset composition following 8 days of differentiation of T_N_ cells. **(C)** CD8^+^ T cell subset distribution within PBMCs population before sorting. T cell subset distribution (T_N_, T_SCM_, T_CM_ and T_EM_) is shown in percentages before sorting.

Supplementary Figure 2 Transcriptomic characterization of CD8^+^ T cell subset identities. **(A)** Enrichment of subset signatures in public bulk RNA sequencing datasets. **(B)** Enrichment of subset signatures in public single-cell RNA sequencing data from Chu et al., 2023 (50). **(C)** Expression of selected T cell markers. **(D)** Overview plot of the three most significant pathways upregulated in each single subset (related to **Figure 1E**).

Supplementary Figure 3 Expression of genes related to glycolysis. Key glycolytic enzymes such as *ENO1*, *HK1*, *PFK*, and *PK* are downregulated in T_N_ cells. Genes were retrieved from MetabolicAtlas.

Supplementary Figure 4 Abundance ratios of selected metabolites. **(A)** Ratio of fumarate to succinate (fum/succ). **(B)** Ratio of glycine to serine (gly/ser). **(C)** Ratio of oxidized to reduced glutathione (gssg/gsh). **(D)** Ratio of NADH to NAD+ (nadh/nad+). **(E)** Ratio of NADH to NAD+ (nadh/nad+) in effector and exhausted cells. **(F)** Ratio of fumarate to succinate (fum/succ) in effector and exhausted cells.

Supplementary Figure 5 Expression of genes in the TCA cycle. Downregulation of multiple TCA cycle enzymes (retrieved from MitoCarta) in T_N_ cells.

Supplementary Figure 6 Expression of mitochondrial NADH shuttles. Expression of genes of the malate-aspartate shuttle (*MDH1*, *MDH2*, *GOT2*, *SLC25A11*, *SLC25A12*, *SLC25A13*), citrate-malate shuttle (*MDH1*, *MDH2*, *SLC25A1*, *ACLY*), glycerol-3-phosphate shuttle (*GPD1*, *GPD2*) and proline cycle (*ALDH18A1*, *PYCR1*, *PYCR2*, *PYCR3*, *PRODH*, *PRODH2*, *ALDH4A1*).

Supplementary Figure 7 Expression of nutrient transporters. Expression of genes associated with GO term “L−amino acid transmembrane transporter activity”.

Supplementary Figure 8 OXPHOS subunit expression. Expression of genes coding for subunits of complexes involved in oxidative phosphorylation as defined in MitoCarta.

Supplementary Figure 9 Mitochondrial gene expression. Mitochondrial abundance calculated from the expression genes coding for selected mitochondrially localized proteins, tested for differences using two-sided t-tests.

Supplementary Table 1 Differentially expressed genes in differentiation and exhaustion.

Supplementary Table 2 Gene set over-representation analysis of gene clusters in differentiation.

Supplementary Table 3 Metabolomics results (differentiation).

Supplementary Table 4 Expression of selected genes in public data.

Supplementary Table 5 Gene set enrichment analysis (exhaustion).

Supplementary Table 6 Metabolomics results (exhaustion).

Supplementary Table 7 List of Reagents.
