# Supplementary figures and images for: 13C tracer analysis reveals the landscape of metabolic checkpoints in human CD8+ T cell differentiation and exhaustion
Source: Front Immunol. 2023 Oct 19;14:1267816. doi: 10.3389/fimmu.2023.1267816 (PMC10620935; doi:10.3389/fimmu.2023.1267816)

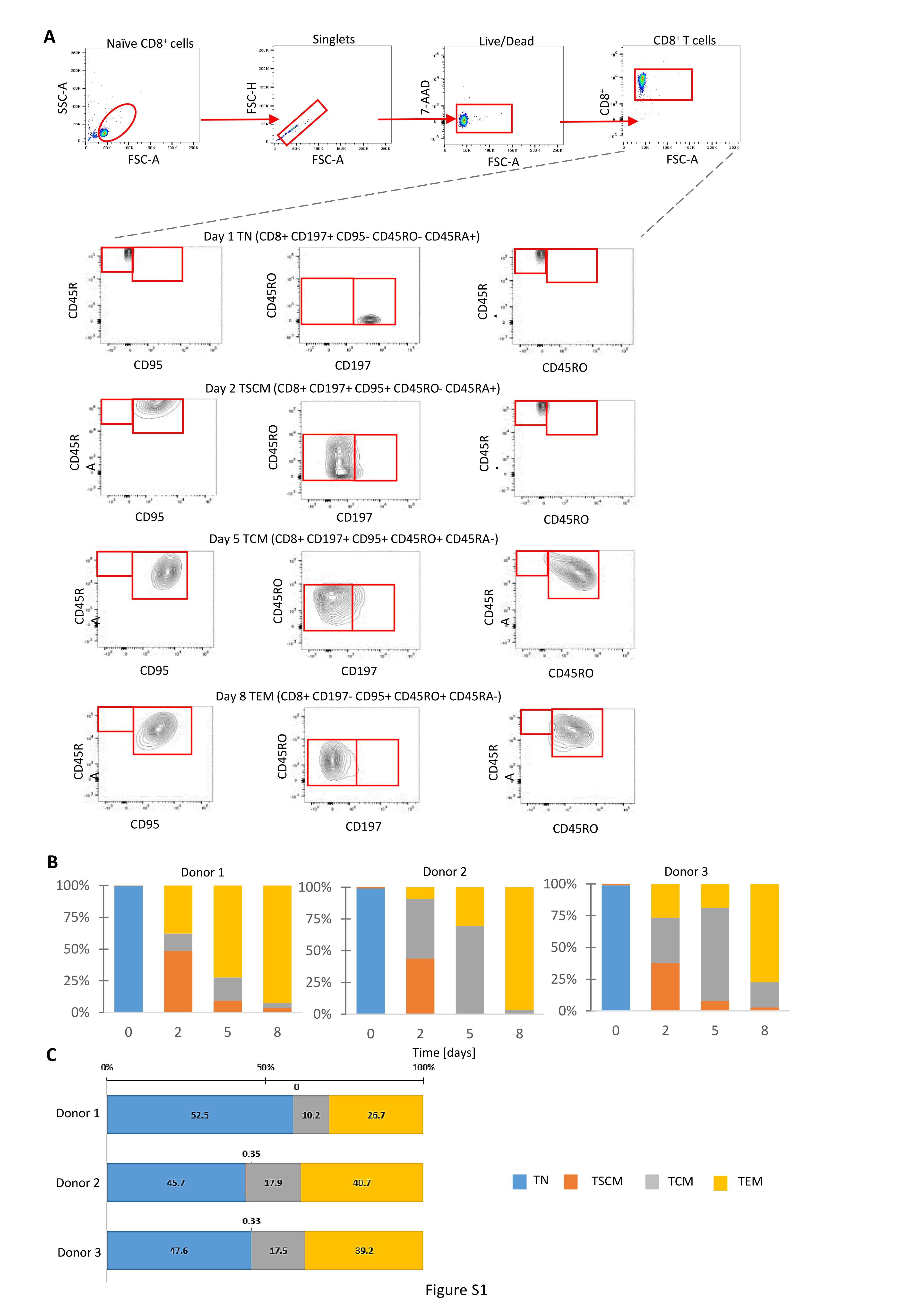

Supplement: Supplementary Figure 1 — (A) Flow Cytometry gating strategy to identify differentiation stages of naïve CD8+ T cells. Naive CD8+ T cells were stained and after doublet and dead cell removal by using forward scatter height (FSC-H) and area (FSC-A) and 7-AAD staining, CD8+ T cell population was further gated into different subsets during the course of 8 days of differentiation (exemplarily plots of Donor 1). Differentiation stages were defined based on the differential expression of CD95, CD45RA, CD45RO and CD197 on CD8+ T cells. TN (naïve T cells, CD8+ CD197+ CD95- CD45RO- CD45RA+); TSCM (stem cell memory T cells, CD8+ CD197+ CD95+ CD45RO- CD45RA+); TCM (central memory T cells, CD8+ CD197+ CD95+ CD45RO+ CD45RA-); TEM (effector memory T cells, CD8+ CD197- CD95+ CD45RO+ CD45RA-). (B) Differentiation dynamics. Changes in subset composition following 8 days of differentiation of TN cells. (C) CD8+ T cell subset distribution within PBMCs population before sorting. T cell subset distribution (TN, TSCM, TCM and TEM) is shown in percentages before sorting. [file Image_1.jpeg]

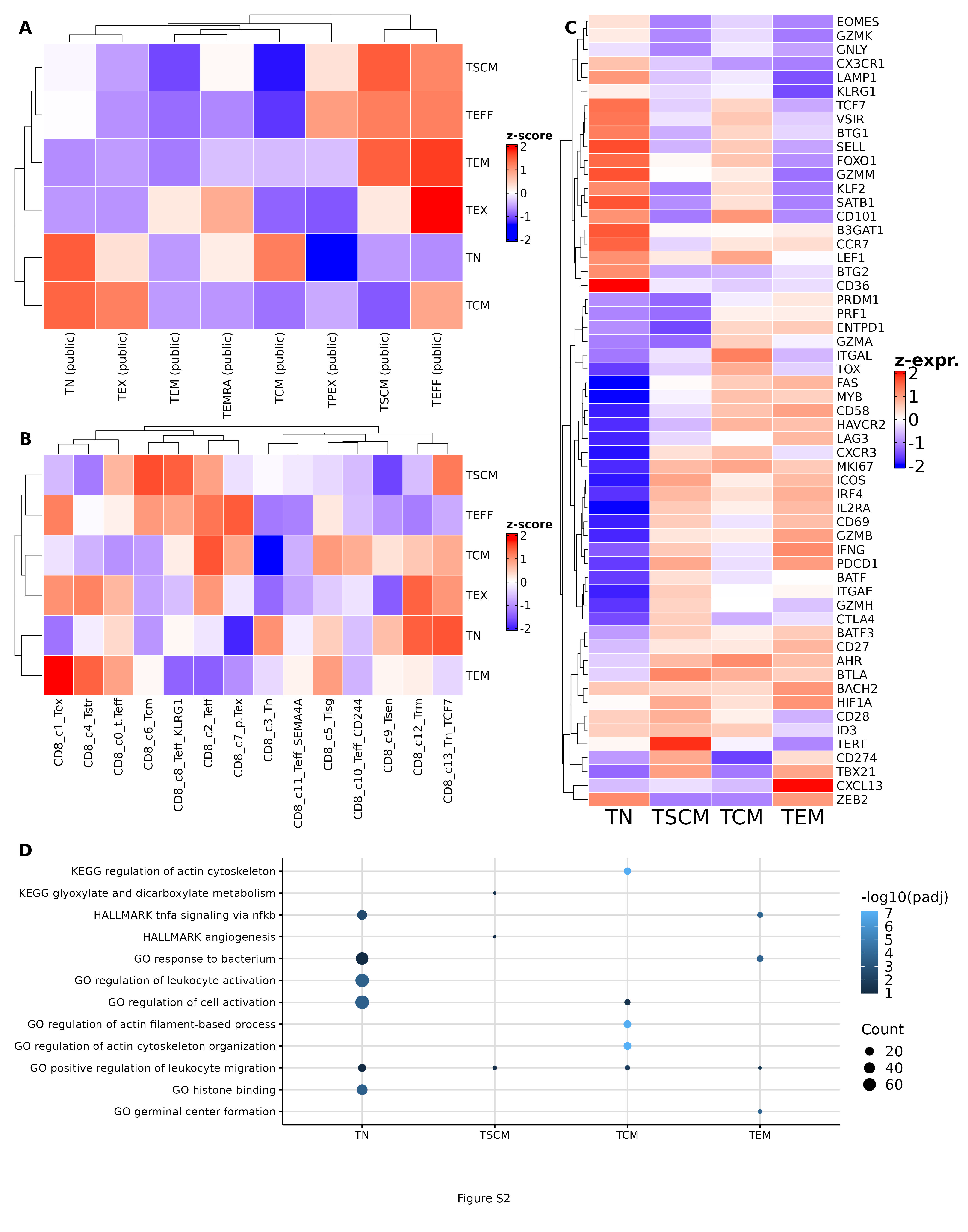

Supplement: Supplementary Figure 2 — Transcriptomic characterization of CD8+ T cell subset identities. (A) Enrichment of subset signatures in public bulk RNA sequencing datasets. (B) Enrichment of subset signatures in public single-cell RNA sequencing data from Chu et al., 2023 (50). (C) Expression of selected T cell markers. (D) Overview plot of the three most significant pathways upregulated in each single subset (related to Figure 1E ). [file Image_2.jpeg]

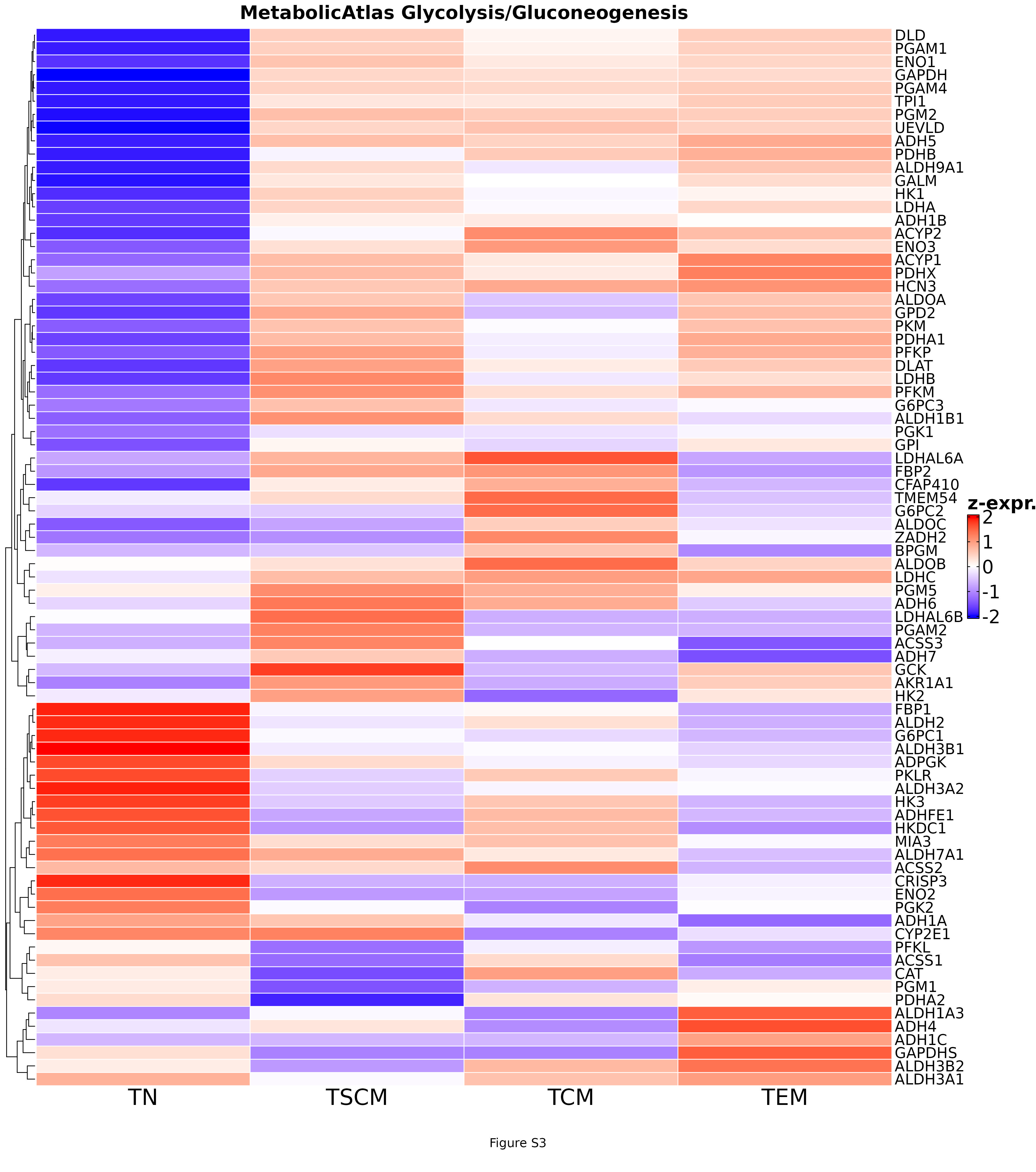

Supplement: Supplementary Figure 3 — Expression of genes related to glycolysis. Key glycolytic enzymes such as ENO1, HK1, PFK, and PK are downregulated in TN cells. Genes were retrieved from MetabolicAtlas. [file Image_3.jpeg]

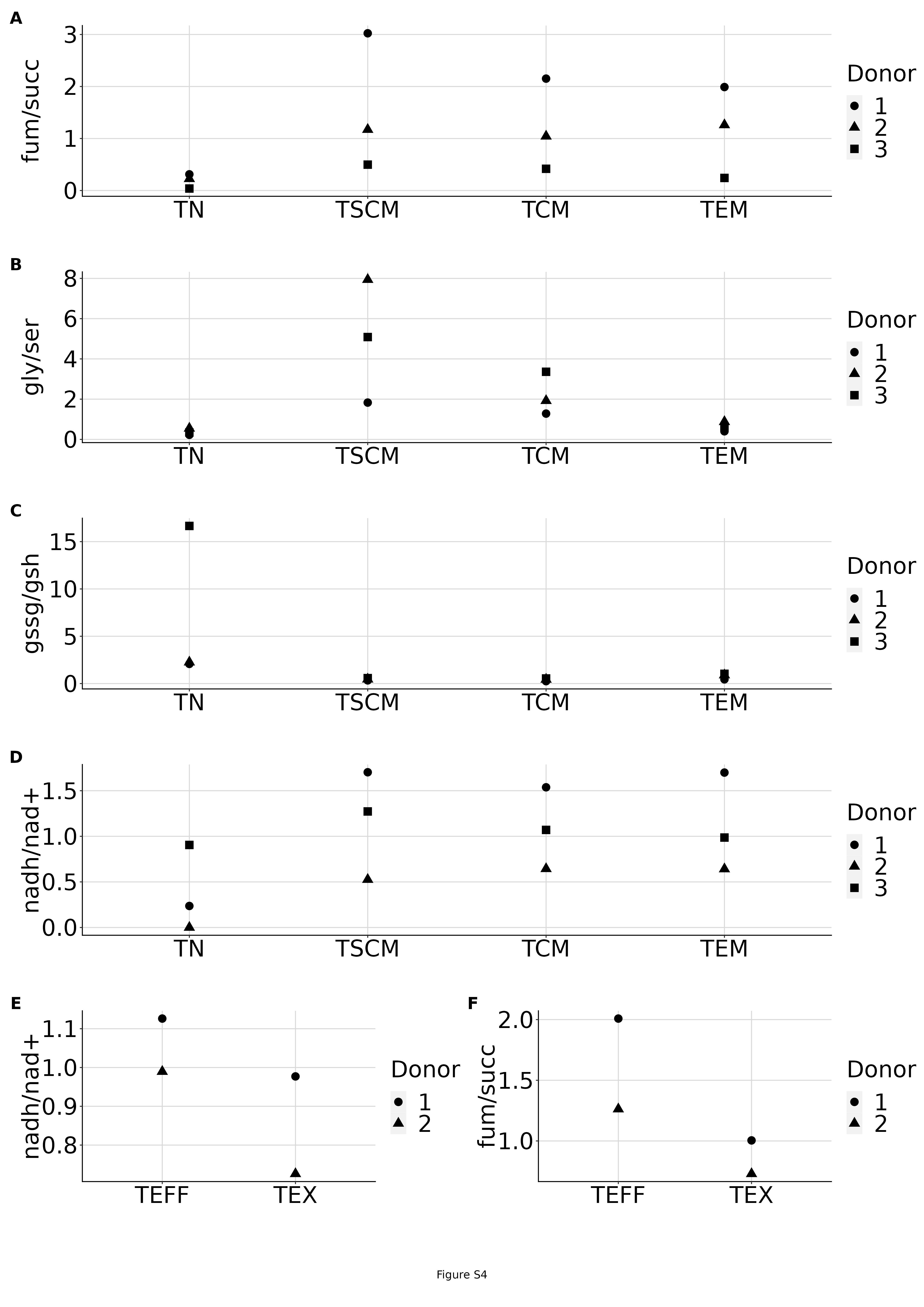

Supplement: Supplementary Figure 4 — Abundance ratios of selected metabolites. (A) Ratio of fumarate to succinate (fum/succ). (B) Ratio of glycine to serine (gly/ser). (C) Ratio of oxidized to reduced glutathione (gssg/gsh). (D) Ratio of NADH to NAD+ (nadh/nad+). (E) Ratio of NADH to NAD+ (nadh/nad+) in effector and exhausted cells. (F) Ratio of fumarate to succinate (fum/succ) in effector and exhausted cells. [file Image_4.jpeg]

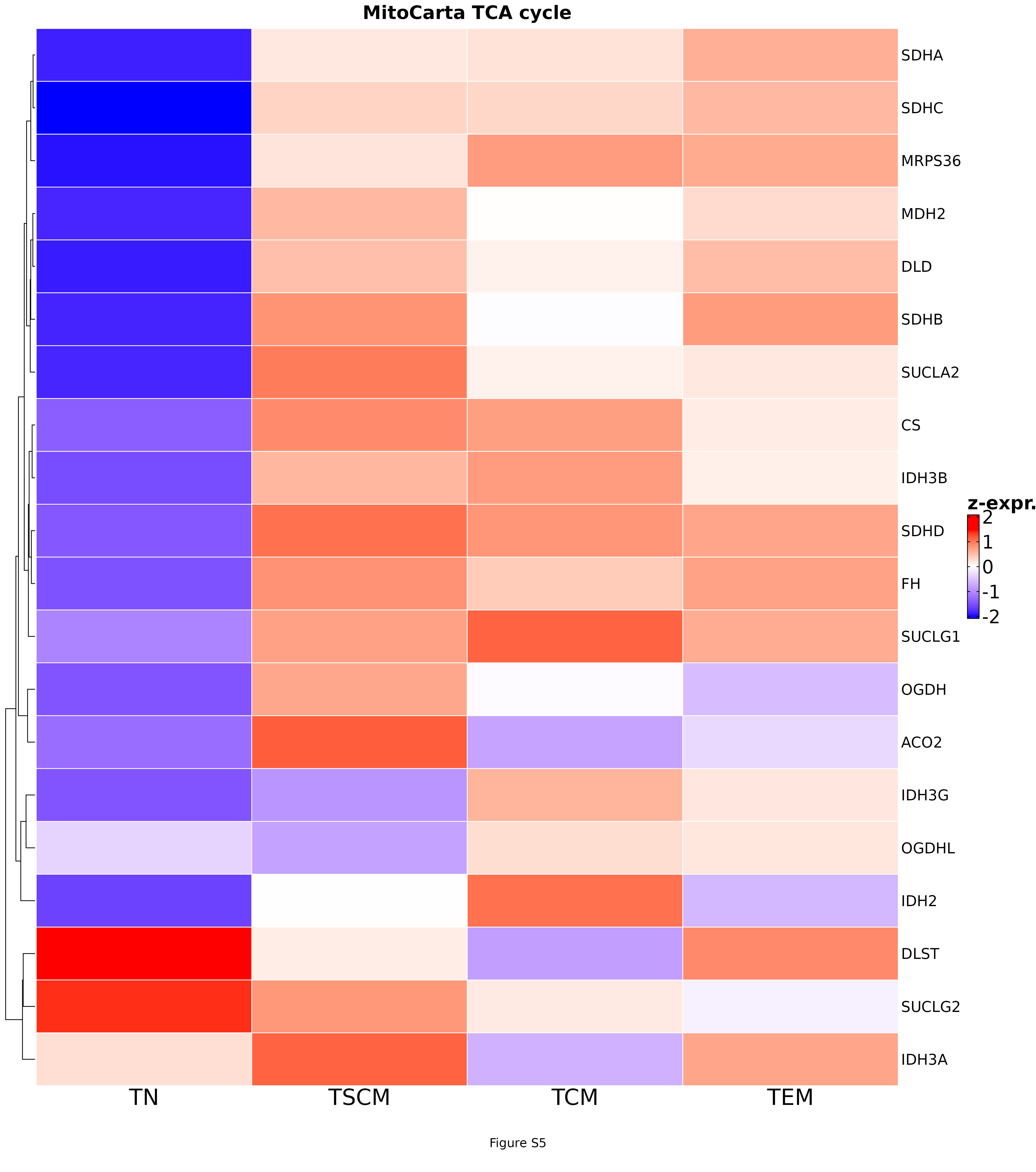

Supplement: Supplementary Figure 5 — Expression of genes in the TCA cycle. Downregulation of multiple TCA cycle enzymes (retrieved from MitoCarta) in TN cells. [file Image_5.jpeg]

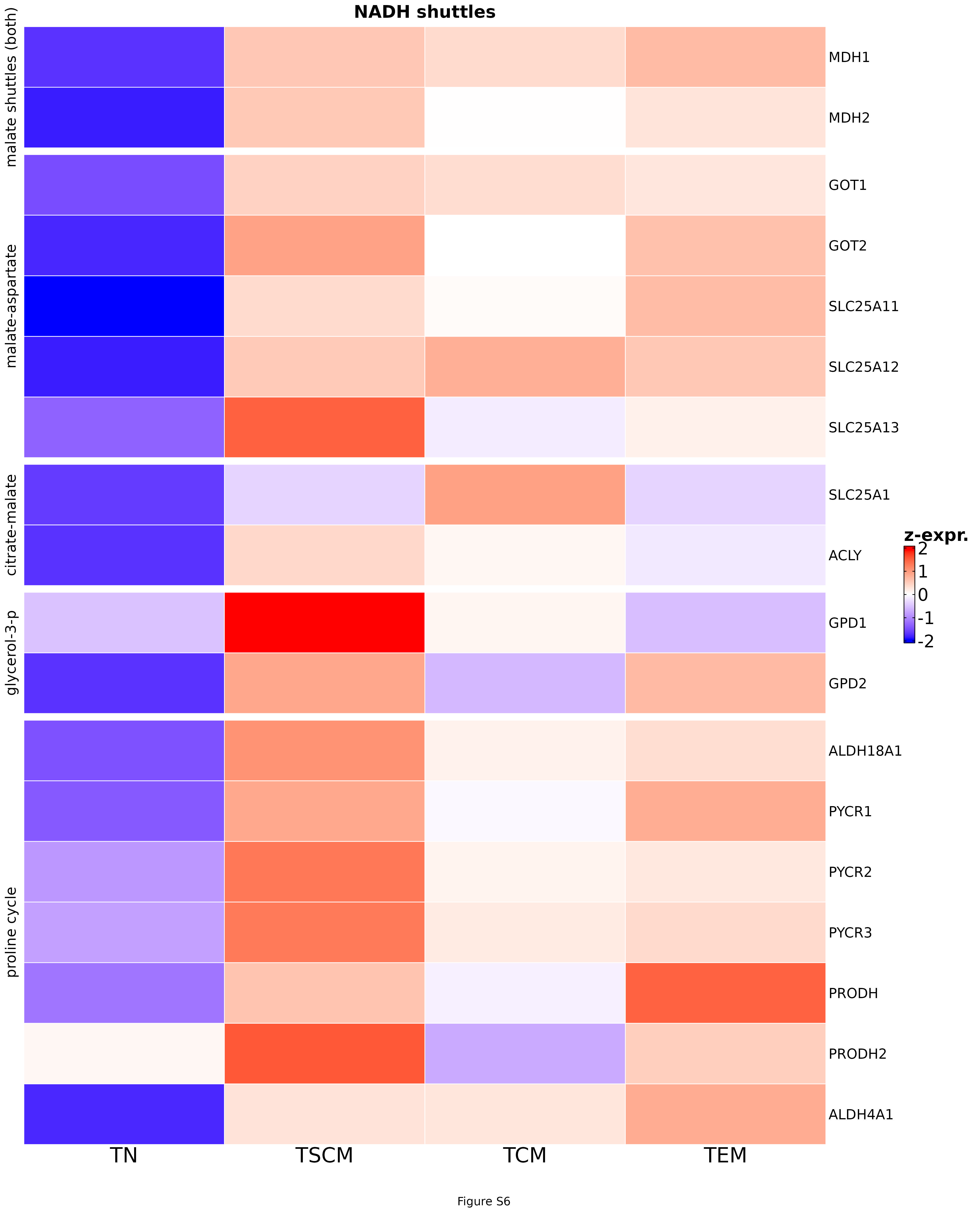

Supplement: Supplementary Figure 6 — Expression of mitochondrial NADH shuttles. Expression of genes of the malate-aspartate shuttle (MDH1, MDH2, GOT2, SLC25A11, SLC25A12, SLC25A13), citrate-malate shuttle (MDH1, MDH2, SLC25A1, ACLY), glycerol-3-phosphate shuttle (GPD1, GPD2) and proline cycle (ALDH18A1, PYCR1, PYCR2, PYCR3, PRODH, PRODH2, ALDH4A1). [file Image_6.jpeg]

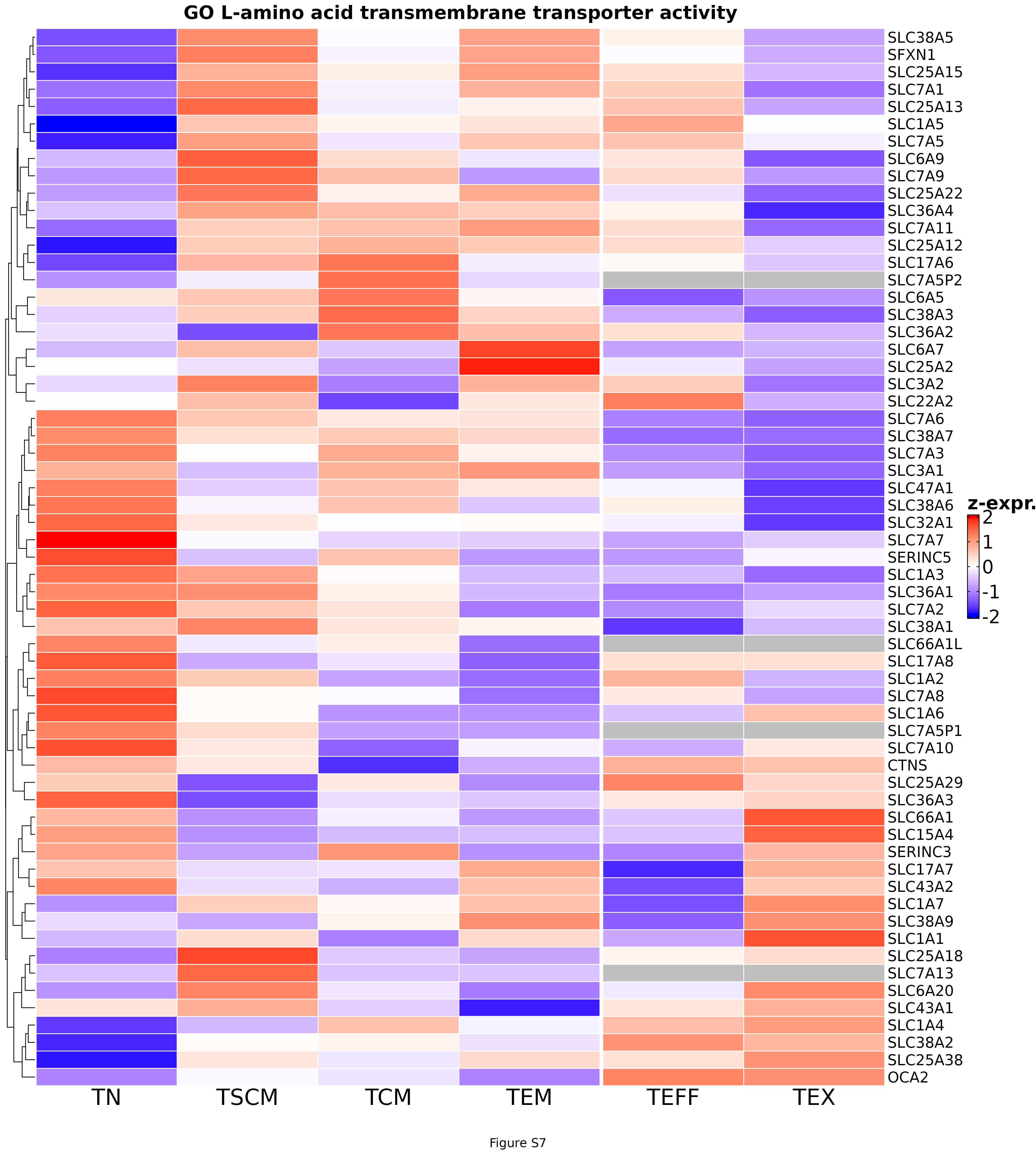

Supplement: Supplementary Figure 7 — Expression of nutrient transporters. Expression of genes associated with GO term “L−amino acid transmembrane transporter activity”. [file Image_7.jpeg]

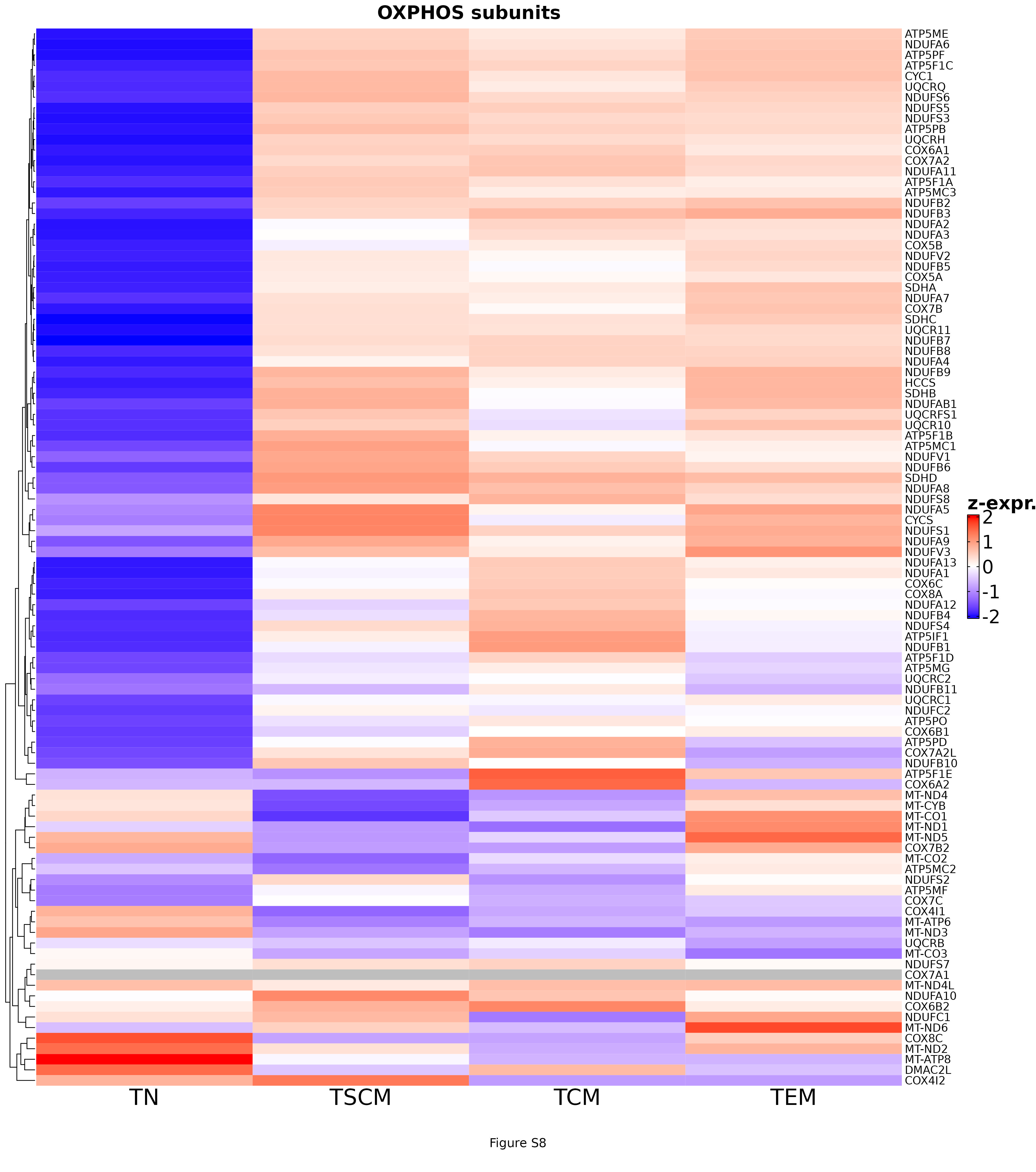

Supplement: Supplementary Figure 8 — OXPHOS subunit expression. Expression of genes coding for subunits of complexes involved in oxidative phosphorylation as defined in MitoCarta. [file Image_8.jpeg]

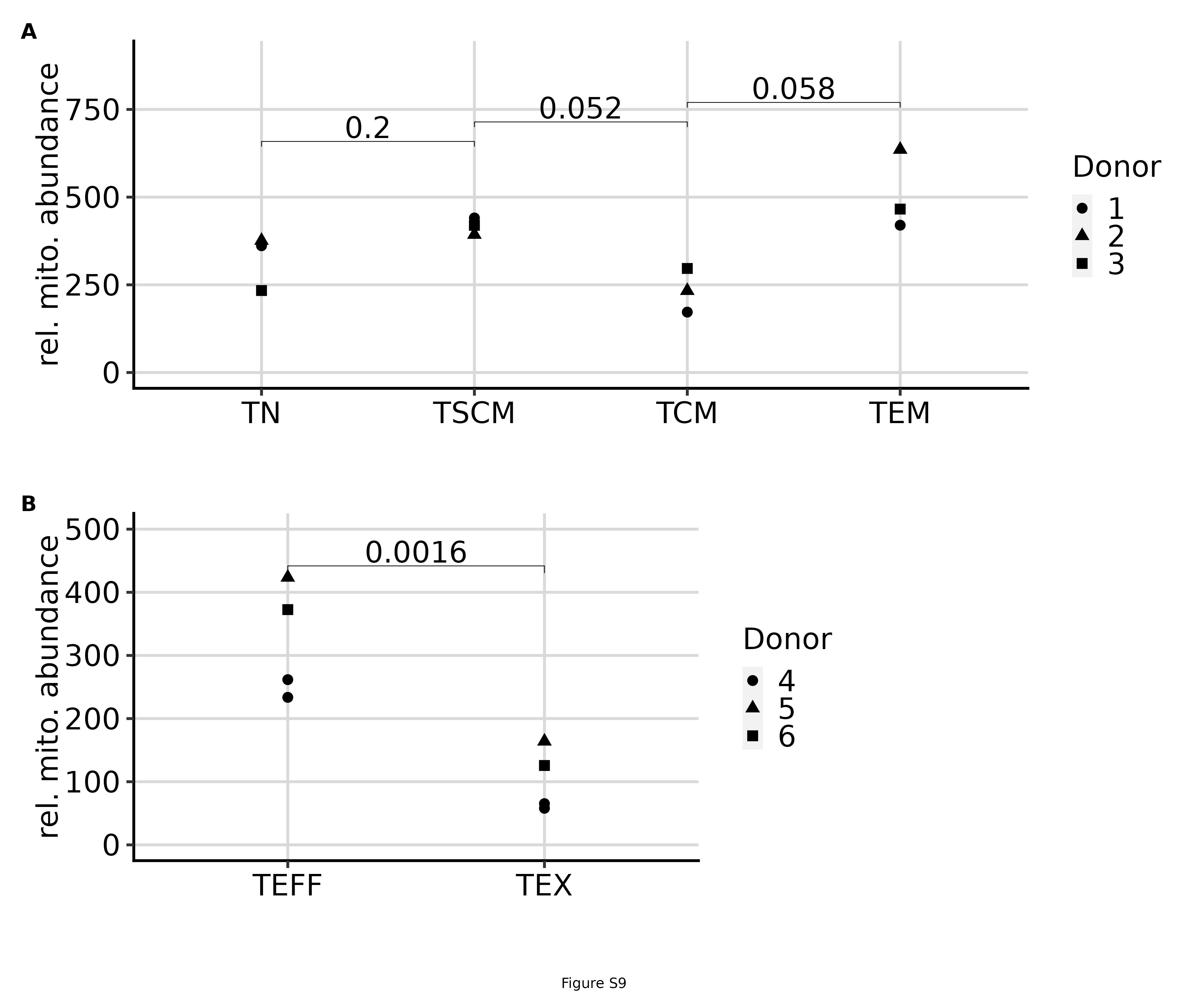

Supplement: Supplementary Figure 9 — Mitochondrial gene expression. Mitochondrial abundance calculated from the expression genes coding for selected mitochondrially localized proteins, tested for differences using two-sided t-tests. [file Image_9.jpeg]
